# Social tipping dynamics for stabilizing Earth’s climate by 2050

**DOI:** 10.1073/pnas.1900577117

**Published:** 2020-01-21

**Authors:** Ilona M. Otto, Jonathan F. Donges, Roger Cremades, Avit Bhowmik, Richard J. Hewitt, Wolfgang Lucht, Johan Rockström, Franziska Allerberger, Mark McCaffrey, Sylvanus S. P. Doe, Alex Lenferna, Nerea Morán, Detlef P. van Vuuren, Hans Joachim Schellnhuber

**Affiliations:** ^a^Earth System Analysis, Potsdam Institute for Climate Impact Research, Member of the Leibniz Association, 14473 Potsdam, Germany;; ^b^Stockholm Resilience Centre, Stockholm University, 11419 Stockholm, Sweden;; ^c^Climate Service Center Germany (GERICS), 20095 Hamburg, Germany;; ^d^Risk and Environmental Studies, Karlstad University, SE 651 88 Karlstad, Sweden;; ^e^Information and Computational Sciences Group, James Hutton Institute, Craigiebuckler, Aberdeen AB15 8QH, Scotland, United Kingdom;; ^f^Observatorio para una Cultura del Territorio, 28012 Madrid, Spain;; ^g^Department of Geography, Humboldt University, 10099 Berlin, Germany;; ^h^Integrative Research Institute on Transformations of Human–Environment Systems, Humboldt University, 10099 Berlin, Germany;; ^i^Department of Geography, University of Innsbruck, 6020 Innsbruck, Austria;; ^j^UN Climate Change community for Education, Communication and Outreach Stakeholders (ECOS), 3046 Kisbágyon, Hungary;; ^k^GeoSustainability Consulting, Adabraka-Accra, Ghana;; ^l^Department of Philosophy, University of Washington, Seattle, WA 98195-3350;; ^m^Germinando Sociedad Cooperativa Madrid, 28012 Madrid, Spain;; ^n^Foro de Transiciones, 28011 Madrid, Spain;; ^o^Climate, Air and Energy, PBL Netherlands Environmental Agency, 2594 AV Den Haag, The Netherlands;; ^p^Copernicus Institute, Utrecht University, 3584 CB Utrecht, The Netherlands;; ^q^Department of Earth System Science, School of Science, Tsinghua University, Haidian District, Beijing 100084, People’s Republic of China

**Keywords:** climate change, Paris Agreement, decarbonization, social tipping elements, social tipping interventions

## Abstract

Safely achieving the goals of the Paris Climate Agreement requires a worldwide transformation to carbon-neutral societies within the next 30 y. Accelerated technological progress and policy implementations are required to deliver emissions reductions at rates sufficiently fast to avoid crossing dangerous tipping points in the Earth’s climate system. Here, we discuss and evaluate the potential of social tipping interventions (STIs) that can activate contagious processes of rapidly spreading technologies, behaviors, social norms, and structural reorganization within their functional domains that we refer to as social tipping elements (STEs). STEs are subdomains of the planetary socioeconomic system where the required disruptive change may take place and lead to a sufficiently fast reduction in anthropogenic greenhouse gas emissions. The results are based on online expert elicitation, a subsequent expert workshop, and a literature review. The STIs that could trigger the tipping of STE subsystems include 1) removing fossil-fuel subsidies and incentivizing decentralized energy generation (STE1, energy production and storage systems), 2) building carbon-neutral cities (STE2, human settlements), 3) divesting from assets linked to fossil fuels (STE3, financial markets), 4) revealing the moral implications of fossil fuels (STE4, norms and value systems), 5) strengthening climate education and engagement (STE5, education system), and 6) disclosing information on greenhouse gas emissions (STE6, information feedbacks). Our research reveals important areas of focus for larger-scale empirical and modeling efforts to better understand the potentials of harnessing social tipping dynamics for climate change mitigation.

## Significance

Achieving a rapid global decarbonization to stabilize the climate critically depends on activating contagious and fast-spreading processes of social and technological change within the next few years. Drawing on expert elicitation, an expert workshop, and a review of literature, which provides a comprehensive analysis on this topic, we propose concrete interventions to induce positive social tipping dynamics and a rapid global transformation to carbon-neutral societies. These social tipping interventions comprise removing fossil-fuel subsidies and incentivizing decentralized energy generation, building carbon-neutral cities, divesting from assets linked to fossil fuels, revealing the moral implications of fossil fuels, strengthening climate education and engagement, and disclosing greenhouse gas emissions information.

**Fig. 1.**
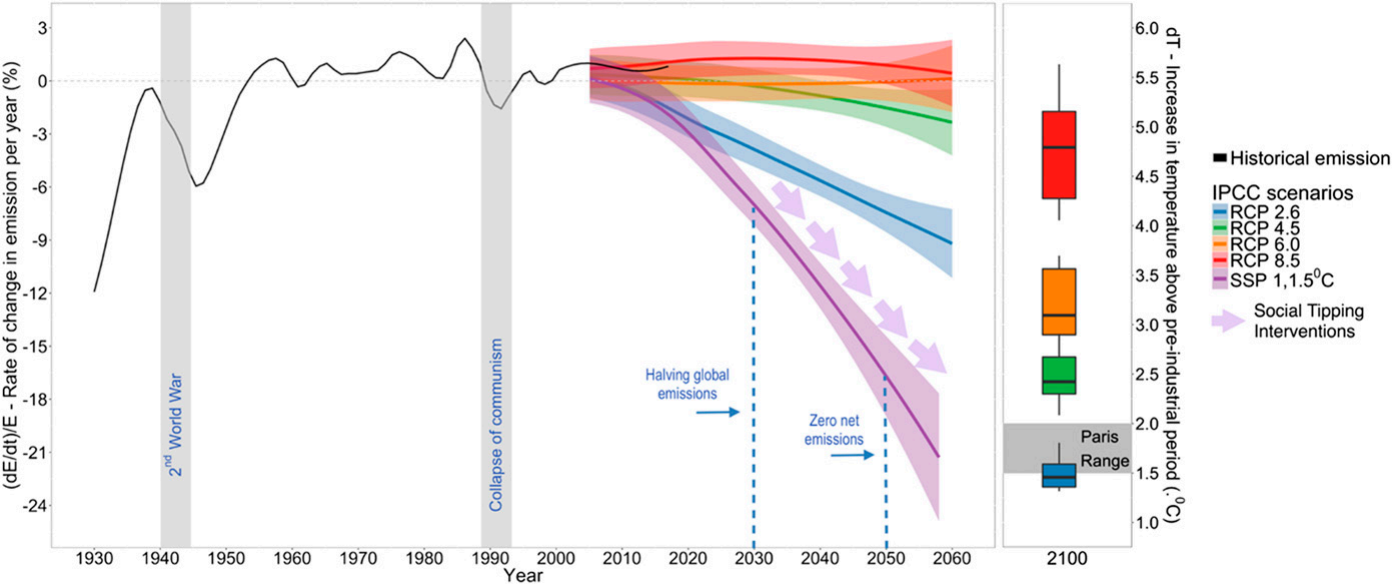
The rate of change in annual greenhouse gas emissions required for net decarbonization. Social tipping dynamics in the context of the representative concentration pathways (RCPs) of the Intergovernmental Panel on Climate Change (IPCC) and the Paris Agreement. *Left* and *Right* exhibit the rate of change in CO_2_ emission per year between 1930 and 2060, and the increase in global mean temperature by 2100 relative to the preindustrial period, respectively, under the four RCPs. The transition to a new net decarbonized state requires shifting from an incremental rise in emissions of 0 to 2% per year to nonlinear decline at the rate of 7% per year and more (6). The figure was created using the RCP emission projections (153) and Coupled Model Intercomparison Project 5 (CMIP5) temperature projections (154).

Preventing dangerous climate change and its devastating consequences is a defining task for humanity (1, 2). It is also an indispensable prerequisite for achieving sustainable development (3, 4). Limiting global warming to 1.5 °C as stipulated in the Paris Climate Agreement (5) scientifically implies a complete net decarbonization of the world’s energy and transport systems, industrial production, and land use by the middle of this century. In their “roadmap for rapid decarbonization,” Rockström et al. (6) highlight that rapid increase of the share of zero-carbon energy within the global energy system would be needed to achieve this objective, likely alongside a considerable strengthening of terrestrial carbon sinks. In one scenario, the zero-carbon share of the energy system doubles every 5 to 7 y for the next several decades (6). Carbon emissions that are currently still on the rise at rates of 0 to 2% per year, despite decades-long efforts in international climate negotiations, would thereby need to pivot to a rapid decline of ultimately 7% per year and more. These emission reduction rates would surpass by far even those experienced only during periods of massive socioeconomic crisis in the 20th century, such as World War II and the collapse of communism (Fig. 1).

Here, the historically decisive question is whether and how such rapid rates of deployment can be collectively achieved. Current deployment rates of low-carbon energy sources are compatible with the required shift but when scaled up are expected to encounter considerable resistance due to the rigidities inherent in political and economic decision making (7, 8), as well as new technological demands (9, 10). Although an increasing number of countries have already introduced or are committed to introducing carbon pricing, the initiatives covered by carbon pricing included only 15% of global greenhouse gas emissions in 2017 (11) and have so far driven only marginal emission reductions (12). It is increasingly recognized that business-as-usual technological progress and carbon pricing alone are not likely to lead to rapid and deep reductions in greenhouse gas emissions (13).

At the same time, there is evidence from various scientific fields demonstrating that rapid rates of change can be observed under certain critical conditions in natural (14–16), socioeconomic (17–20) and social-ecological systems (SESs) (21, 22). Increasing attention is being given to the concept of tipping dynamics as a nonlinear mechanism behind such disruptive system changes. Based on a review on social-ecological tipping points research, Milkoreit et al. (23) propose a common definition of social tipping points (STPs) as points “within an SES at which a small quantitative change inevitably triggers a non-linear change in the social component of the SES, driven by self-reinforcing positive-feedback mechanisms, that inevitably and often irreversibly lead to a qualitatively different state of the social system.” There are historical examples of dynamic social spreading effects leading to a large self-amplification of small interventions: For example, the writings of one man, Martin Luther, injected through newly available printing technology into a public ready for such change, triggered the worldwide establishment of Protestant churches (24). An example in the field of climate policy is the introduction of tariffs, subsidies, and mandates to incentivize the growth of renewable energy production. This has led to a substantial system response in the form of mutually reinforcing market growth and exponential technology cost improvement (25, 26).

In this paper, we examine a number of potential “social tipping elements” (STEs) for decarbonization (27, 28) that represent specific subdomains of the planetary social-economic system. Tipping of these subsystems could be triggered by “social tipping interventions” (STIs) that could contribute to rapid transition of the world system into a state of net zero anthropogenic greenhouse gas emissions. The results reported in this study are based on an online expert survey, an expert workshop, and an extensive literature review (*SI Appendix*).

Our results complement the existing shared socioeconomic pathways (SSPs) that are used alongside the representative concentration pathways (RCPs) to analyze the feedbacks between climate change and socioeconomic factors, such as world population growth, economic development, and technological progress (29). Our results could be useful for exploring possible transformative pathways leading to scenarios that reach net zero emissions by 2050 (30).

## Defining STEs and STIs Relevant for Decarbonization Transformation

Various types of tipping processes can be differentiated in the literature. Many authors refer to critical thresholds (16, 28), a notion closely related to the metaphor of a “butterfly effect” (31, 32). Other processes related to tipping dynamics include metamorphosis, where a rapid loss of structures of one sort occurs simultaneously with the development of new structures (33), as well as cascades driven by positive feedbacks in processes occurring simultaneously at smaller scales (34).

The social tipping dynamics of interest for this study are typically manifested as spreading processes in complex social networks (35, 36) of behaviors, opinions, knowledge, technologies, and social norms (37, 38), including spreading processes of structural change and reorganization (34). These spreading processes resemble contagious dynamics observed in epidemiology that spread through social networks (39). Once triggered, such processes can be irreversible and difficult to stop. Similar contagious dynamics have been observed in human behavior (35, 36), for example in assaultive violence (39), participation in social movements (40), or health-related behaviors and traits (36), such as smoking or obesity (41, 42).

We understand STEs as functional subsystems of the planetary-scale World–Earth system (43) consisting of interacting biophysical subsystems of the Earth, and the social, cultural, economic, and technological subsystems of the world of human societies (43, 44). Potential STEs share one defining characteristic: A small change or intervention in the subsystem can lead to large changes at the macroscopic level (23) and drive the World–Earth system into a new basin of attraction, making the transition difficult to reverse (20). Exact quantifications of the relationship between big and small are, however, rare, as are empirical examples (Table 1). For the combination of big interventions and big effects, there are currently no convincing examples; however, the potential use solar radiation management geoengineering in the future would fall into this category. Finally, some changes in the World–Earth system might be driven by nonhuman and unintentional forces (e.g., a sufficiently large meteorite hitting the Earth or a disease outbreak), while others might be driven by conscious interventions of human agency (45).

**Table 1.** Illustrative examples of intervention-and-effect relationships in the context of climate change mitigation

Intervention types	Small effect	Big effect
Small intervention	An incremental change, e.g., a town mitigation plan (157)	A tipping effect, e.g., feed-in tariffs in the German “Energiewende” (158)
Big intervention	Inefficient interventions, e.g., the implementation of the European Carbon Emission Trading Scheme leading to a marginal reduction of greenhouse gas emissions due to leakage effects (159)	An elephant effect, e.g., reducing the Earth’s carbon burden by means of solar radiation management geoengineering (160)

Tipping processes might be analyzed as a function of change in a suitably selected forcing variable or control parameter (15, 27). The pertinent World–Earth system features such as the anthropogenic carbon emissions are commonly the product of complex interactions of multiple drivers. These factor can, however, in some cases be combined into a single dominant control parameter.

**Fig. 2.**
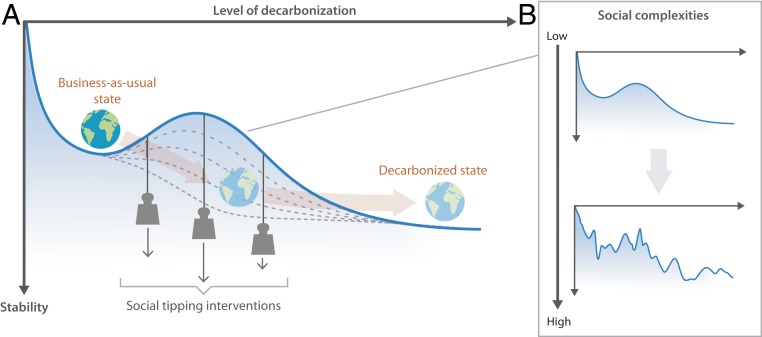
The concept of decarbonization transformation as social tipping dynamics. As illustrated in *A* by an abstract stability landscape (155), the world’s socioeconomic system today is trapped in a valley where it still depends heavily on burning fossil fuels, leading to high rates of greenhouse gas (GHG) emissions. STIs have the potential to erode the barrier through triggering social tipping dynamics in different sectors (Fig. 3) and thus paving the way for rapid transformative change. Uncertainties and complexities inherent in the many dimensions of human societies beyond their level of decarbonization (46) can be envisioned as forming a rougher stability landscape featuring multiple attracting states and a larger number of barriers that need to be eroded or overcome (*B*). This inherent “social noise” may complicate transformative change but could also accelerate it by means of dynamical phenomena such as stochastic resonance (156).

In this study, we identify a subsystem of the World–Earth system as a STE relevant for decarbonization transformation if it fulfils the following criteria:

C1. A set of parameters or drivers controlling its state can be described by a combined control parameter that after crossing a critical threshold (the STP) by a small amount influences a crucial system feature of relevance (here the rate of anthropogenic greenhouse gas emissions) leading to a qualitative change in the system after a reference time has passed allowing for the emergence of the effect (15).
C2. It is possible to differentiate particular human interventions leading to the small change in the control parameter that has a big effect on the crucial system feature, which will be referred to as the STI (Fig. 2).

Established social systems, including their infrastructures, while they may partly be open to change, tend also to possess self-stabilizing mechanisms that oppose change, be it through infrastructural inertia due to investment cycles or cultural or political inertia due to deeply held traditions or power structures all representing aspects of social complexities (Fig. 2 and refs. 46 and 47). For this reason, a cumulation of effects due to social contagion, repetitive nudging, or direct intervention can lead to social tipping dynamics (48). Starting points for such cumulations of effects are here called STIs. Naturally, their existence, nature, and point of departure are a function of the cumulated history of the respective social system and, in that sense, STIs and social tipping dynamics are path dependent.

Following Rockström et al. (6), in order to achieve the Paris Climate Agreement’s goals and to avoid higher levels of global warming at the end of this century that would imply crossing dangerous tipping points in the Earth’s climate system (27), global anthropogenic carbon emissions would need to be halved every decade, achieving a peak in 2020 and then steadily decreasing to reach net zero emissions by 2050. Achieving net zero global emissions around 2050 is necessary for there to be a significant probability of limiting global warming to 1.5 °C by the end of the century (1). To ensure that the social tipping dynamics identified in this study are compatible with these constraints, we impose the following filtering criteria:

F1. The time needed to trigger the tipping should not exceed ∼15 y, and the time needed to observe a qualitative change at the whole system level should not exceed ∼30 y (Fig. 1).
F2. Since abrupt social changes have historically often been associated with social unrest, war, or even collapse (49), human intervention and its foreseeable effects should here be explicitly compatible with the Sustainable Development Goals (50), in the sense of positive social tipping dynamics (34).

Finally, due to the networked and multilevel character of the social system (51), we also ask about the feedback mechanisms connecting and potentially mutually reinforcing the identified candidates for STEs and STIs.

## Results

### Candidates for STEs from Expert Elicitation.

Both natural and social systems are characterized by a high level of complexity and are linked by coevolutionary dynamics (52). Isolating the elements of such systems is difficult. Although we provided our respondents with a written definition of a STE, most of the online survey participants referred to what we define as STIs. On the basis of the responses, 12 groups of candidates for STEs could be identified, each referring to a distinctive control parameter (Table 2). The critical threshold of the control parameter needed to be crossed in order to trigger the tipping process was in most of groups not quantified by the experts but described qualitatively. The STP was often referred to as the point when a certain belief, behavior, or technology, spreads from a minor tendency to a major practice. Documented instances of technology and business solutions show that a 17 to 20% market or population share can be sufficient to cross the tipping point and scale up to become the dominant pattern (53). Some authors, however, argue that it must be the “right” share of population, including well-connected influential people, trendsetters, and other types of social leaders with a high degree of agency (38, 54). In other cases, the experts referred to the STP that would be achieved if the price of fossil-fuel–free products and services falls below that of those products and services based on fossil fuels. Table 2 presents an overview of expert elicitation results.

**Table 2.** The candidates for social tipping elements for rapid decarbonization identified by expert elicitation

Candidates for social tipping elements	Key actors able to influence the control parameter	Main control parameter	Examples of interventions	Critical threshold in the control parameter
Climate policy enforcement	International agencies, national and local governments, political elites, industry, NGOs, business, the public	The number of regulations restricting the use of fossil fuels	A global environmental court; producer responsibility and circular economy; limiting the use of fossil fuels sector by sector; banning advertisement of fossil-fuel products; abolishing the trade in fossil fuels	Eliminating the use of fossil fuels from most of sectors and spheres of human life
n* = 42 (20%); Conf.^†^=3				
Information feedback	Scientific community, media, citizen organizations, industry	The share of products and services containing GHG emission information	Adequate information on emissions of products and services; labeling; growing awareness of global risks and health consequences	The GHG emissions information visible for most of products and services
*n* = 37 (17%); Conf.=3				
Financial market	International agencies, national and local governments, financial sector, industry	Market value of fossil-fuel extraction and industry	Carbon taxes and permits; Divesting; reinvesting; national banks warning commercial banks to reduce risk with carbon-intensive investments	The market value decreasing rapidly in comparison with other comparable investments
*n* = 26 (12%); Conf.=3.6				
Energy production and storage	Conventional and green industries, national and local governments, NGOs, public–private partnerships	The relative price of fossil-fuel–free energy production and storage	Cessation of subsidies for fossil-fuel technologies; decentralized and distributed energy generation; renewable energy deployment; community energy hubs; nuclear energy deployment	The price of fossil-fuel–free energy becoming lower than the price of fossil-fuel energy
*n* = 24 (11%); Conf.=3.8				
Knowledge system	Intellectual leaders, scientific community, media	The number of people having worldviews accounting for socioecological complexities	Reconceptualization of economics and valuation measures; convincing narratives of what can be gained from decarbonization; indigenous approaches to nature	The worldviews spreading from the minority to the majority of key actors
n* = 16 (7,7%); Conf.^†^=3.7				
Other technology	Industry, governments, media, agro-industry	Energy demand	Digitalization of the economy; tele-working; e-mobility; artificial meat; multipurpose farm-ponds	Energy demand reduced to a level that can be sustainably produced
*n* = 15 (7%); Conf.=4				
Values and norms	Spiritual leaders, media, young generation, middle class	The perception of fossil fuels as immoral	A new set of moral and ethical codes; revealing the moral implications of fossil fuels, stigmatization of fossil fuels	Spreading from the minority to the majority of key actors
*n* = 12 (6%); Conf.=3				
Human settlements	Industry, city authorities, governments	The demand for fossil-fuel–free technology	Reallocation and redesigning of human settlements; energy independent housing; new building materials; carbon-neutral cities	Fossil-fuel–free technology becoming the first choice in new infrastructure projects
*n* = 10 (5%); Conf.=3.7				
Lifestyles	Food and car industry, writers, wealthy fashionable people, media	Number of people choosing fossil-fuel free products	Vegetarian diets; lower consumption; fossil-fuel free consumption	Spreading from the minority to the majority of the population
*n* = 10 (5%); Conf.=3.7				
Citizenship involvement	Civic and nonprofit organizations, media, the public	Citizenship commitment to climate mitigation	Grassroots organizing resistance; a global network of social movements	From a minor tendency to a global citizen movement
*n* = 7 (3.8%); Conf.=3.1				
Education system	Scientists, teachers, educational ministries	The presence of climate change and relevant concepts in the public education	New educational programs at all levels of public education including climate change, ecological networks, system thinking	The relevant concepts becoming a part of the main curriculum
*n* = 5 (2.4%); Conf.=3.2				
Population control	Political leaders, religious organizations	The number of greenhouse gas emitters	Limiting human population growth	Population decreasing to a number that can be sustainably supported
*n* = 3 (1.4%); Conf.=2.3				

* *n*: The frequency of survey answers is referring to the number of the survey answers refereeing to this topical area and a share (percentage) of total survey answers.

^†^ Conf.: How confident are you that the associated social tipping point is actually going to take place and contribute substantially to a rapid and complete global decarbonization by 2050? 1, Very uncertain; 2, uncertain; 3, rather uncertain, 4, rather confident; 5, confident; 6, very confident.

### Critical Interventions for Inducing Social Tipping Dynamics.

Building upon the results of our expert elicitation, we differentiated six key candidates for STEs and associated STIs for which we were able to find empirical material showing that they fulfill the conditions specified in our definition (as listed in Table 3). These do not necessarily comprise a comprehensive list of “silver bullet” solutions; rather, this is an initial selection that can help in developing more refined socioeconomic rapid transformation pathways and narratives customized at appropriate scales. Below, we present a review of literature on each of the STEs and STIs nominated by the experts. We search for evidence supporting the potential of the interventions to trigger tipping-like changes in their domains leading to a qualitative change at the World–Earth system level; we ask whether critical thresholds in the control parameters can be determined; and finally we begin to examine the interactions and feedbacks among the identified tipping elements.

**Table 3.** Synthesis of the research results on the key candidates for social tipping elements selected by the experts and their associated social tipping interventions

Social tipping element	Social tipping intervention	Control parameter	Key actors	GHG emission reduction potential	Dominant social structure level	Estimated time needed to trigger tipping
STE1: Energy production and storage	STI1.1: Subsidy programs	The relative price of fossil-fuel–free energy	Governments, energy ministries, big energy producers (68)	Up to 21% globally in 1 y (68)	National policy (68)	10 to 20 y (including the policy-formative phase) (161)
	STI1.2: Decentralized energy production		Citizens, communities (73), local governments (162), policy makers (163), energy planners (164)	Up to 100% in power supply (61)	Community/town governance (165)	Less than 10 y (81)
STE2: Human settlements	STI2.2: Carbon-neutral cities	The demand for fossil-fuel–free technology	City administration, citizens, and citizen groups (166)	Reduction by 32% in 14 y (91)	Urban governance (91)	Approximately 10 y (91).
STE3: Financial market	STI3.1: Divestment movement	Profitability of fossil fuel exploitation	Financial investors (96)	26% emissions tied to investments of a large Canadian university (167)	Market exchange, enterprise (98)	Very rapid, could occur within hours (142)
STE4: Norms and values system	STI4.1: Recognition of the immoral character of fossil fuels	The perception of fossil fuels as immoral	Peer groups, environmental organizations, youth, opinion leaders (168–170)	Unprecedented	Informal institutions, enforcement through peer groups (171)	30 to 40 y (172)
STE5: Education system	STI5.1: Climate education and engagement	Climate change and impacts awareness	Teachers, climate educators (117), youth (113)	Up to 30% reduction in 2 y in the emissions of the Italian households included in the study (124)	National policy (173)	10 to 20 y (173)
STE6: Information feedback	STI6.1: Emission information disclosure	The number of products and services disclosing their carbon emissions	The business and producers; governments for setting disclosure guidelines and regulations (174)	Up to 10% reduction of emissions in UK households' grocery consumption in a year (175)	Market, exchange (176); enterprise (177)	A few years (178)

#### STIs in the energy production system.

The technological development in the energy production system is a dominant element of the decarbonization discussions in international institutions (55, 56) and business partnerships (57). The results of our expert elicitation confirm that technology development is likely to play a key role, however, not in the sense of yet-to-be invented technological solutions, but rather in the adaptation of existing carbon-free technology primarily in the power sector and by facilitating a smarter utilization of energy. The main control parameter that drives the adaptation of fossil-fuel–free energy technology is associated with the financial returns of its adoption (58). Our expert group believed that the critical condition needed to trigger the tipping process is the moment when fossil-fuel–free energy production yields higher financial returns than the energy production based on fossil fuels. The empirical data show that this critical threshold is about to be reached; the prices of renewables have dropped sharply in the last few years, and they have already become the cheapest source of energy in many world regions. The average cost of onshore wind dropped by 18%, and offshore wind fell by 28% (59). The costs of photovoltaic modules fell by about 20% with every doubling of cumulative capacity since the 1970s (60) and the key role in reducing the cost of photovoltaics was played by policies that stimulate market growth (26). Optimization modeling shows that renewable energy supplies can potentially supply 100% of human power demand (61), and in theory, rapid transformation to low energy demand is possible (30) and will be cost-effective in the long run (62). However, there are large costs associated with adapting existing infrastructure and supply and demand support services to meet the demands of nondispatchable, volatile renewable sources like wind and solar in electricity generation. The question is whether the cost of transforming the energy infrastructure is worthwhile compared to the cost of inaction. The prioritization of societal preferences in the competition for scarce budgetary resources is influenced by the dominant social values (63).

Our expert group believed that redirecting national subsidy programs to renewables and low-carbon energy sources or removing the subsidies for fossil-fuel technologies are the tipping interventions that are needed for the take-off and diffusion of fossil-fuel–free energy systems. The key actors who have the agency to implement these interventions include national governments and energy ministries, and the response of large energy companies is important. One-third of global industrial greenhouse gas emissions can be linked just to 29 oil and gas companies (64). The International Energy Agency has tracked fossil-fuel subsidies over the last decade and in 2009 estimated that $312bn was spent worldwide in fossil-fuel subsidies, compared to $57bn on renewables in that year (65). By 2015, the gap had narrowed, but the subsidies received by fossil fuels were still more than twice those of renewables (66). Estimates show that a universal phaseout of fossil-fuel subsidies could lower annual carbon emissions by 4.4% (67). Coady et al. (68) argue that eliminating subsidies for fossil fuels would have reduced global carbon emissions in 2013 by 21%.

Furthermore, our expert group believed that the global energy production and storage system can also be radically changed by decentralization of energy production. Since large power stations relying on coal, oil, or gas exploitation are not profitable below a certain threshold of households supplied, decentralized generation systems and transitioning to local power generation might be expected to lead to a virtually complete decarbonization of production systems (69, 70). However, this is also likely to lead to an increase in costs due to the loss of economies of scale (69), and the complexities of integrating variable, distributed power sources (71). This emphasizes the need for decentralized energy generation and demand management to be part of the wider energy systems transformation (72). It has been argued that citizens also have a major role to play as nodes in a smart system capable of facilitating flexible demand management (73). Some authors also warn that meeting current levels of demand (let alone future projected demand) with renewables alone is likely to be extremely difficult (74, 75). Nonetheless, interest in decentralized control of energy systems is growing. Across the Global North, there are a multitude of examples of energy cooperatives and community-driven energy projects (76). Such projects have often found creative ways to overcome limitations imposed by centralized distribution networks, e.g., by using smart technologies to divert excess power for local heating (77), or by bringing municipal supply networks into community ownership (78). They show such initiatives may also spark around the Global South by skipping the “megadevelopment” phase associated with large power stations and massive grid infrastructure expansion. Due to the positive knowledge and technology spillover effects from such decentralized systems, the technology costs are likely to be further reduced with their increased diffusion (79, 80). The time elapsing between the planning phase and actual installation and utilization of decentralized energy generation is reportedly less than 10 y (81). However, existing energy systems and infrastructure are likely to shape the future for decades to come (82).

#### STIs in human settlements.

Direct and indirect emissions from buildings account for almost 20% of all carbon emissions, and we observe an unprecedented scale of global urbanization; each week the global urban population increases by 1.3 million (55). The average life span of buildings is about 50 y (83). Public infrastructure and planning structures can last even longer (50 to 150 y) and play an active role in both climate mitigation and adaptation (84). Modifying building codes for construction and infrastructural projects can actively drive the demand for fossil-fuel–free technologies and are crucial especially for countries in the Global South, where building booms are driving up energy and other resource use (85). An example of a STI in this realm is the creation of large-scale demonstration projects such as carbon-neutral cities. These are important in order to educate the general public and stimulate consumer interest in environmental technologies, accelerating their dissemination and commercialization (85). In addition, local technology clusters create positive spillover effects of lowering the information and transaction costs (86), which can indirectly lead to a reduction in the costs of fossil-fuel–free technologies for energy production and storage. The critical conditions for social tipping in this control parameter would be achieved if the fossil-fuel–free technology became the first choice for new construction and infrastructure projects. There are many new construction materials that not only imply lower emissions but also could actively support carbon sequestration efforts in urban areas. To give an example, constructing a 142-m-high residential building using above 80% laminated timber could lead to sequestrating 21,040 tons CO_2_ and avoiding 50,000 tons CO_2_ emissions otherwise entailed in using standard construction materials such as steel and concrete, which is equivalent to the amount 33,000 cars emit per year (87). In addition, large-scale public infrastructure investments support the emergence of a shared belief in the emerging new social equilibrium that can help individuals coordinate changes and find new focal points (88). The example of the Transition Town Movement that started in 2006 in the United Kingdom and in 2014 spanned over 41 countries shows how local grassroots initiatives can encourage citizens to take direct action toward lowering energy demand and building local resilience despite lack of policy support at national levels (89). Another example includes the Energy Cities Association, whose primary goal is to accelerate the transition to sustainable energy in urban areas in Europe. The Association was created in 1990 and currently represents more than 1,000 towns and cities in 30 countries (90). The evidence from a case study on communities implementing plans for zero emissions shows that these communities were able to reduce their per-capita emissions by 32% in 14 y (91).

#### STIs in the financial system.

The financial crisis in 2008 demonstrated how rapidly changes in the market value of assets in one sector and country can propagate and destabilize the global system of human societies and accelerate changes at the level of individual investment and consumption behavior as well as collective-organizational and policy responses (92). Maintaining global warming below 2 °C implies that 33% of oil, 49% of gas, and 82% of coal resources should not be burned (93). This suggests there might be a risk of a carbon bubble, caused by the financial exposure from stranded assets, which could be driven by policy, technological innovation, or investors’ decisions (94). A growing number of analysts believe a financial bubble is emerging that could burst when investors’ belief in carbon risk reaches a certain threshold (95). Simulations show that just 9% of investors could tip the system, inducing other investors to follow (96). An example of an intervention that can lead to a rapid decline in the control parameter—the value of fossil-fuel assets—is the divestment movement; as it progresses, it results in the reduction of the value of fossil-fuel assets (97). The movement started with a student campaign in 2011 and is quickly expanding to other countries and types of asset owners. The value of investment funds committed to selling off fossil-fuel assets reached $5.2tn in 2016, doubling in just over a year and permeating enterprises in every sector of society, with examples including universities, faith groups, pension funds, and insurance companies (98). Ritchie and Dowlatabadi (94) present model scenarios showing that a major Canadian university could reduce the greenhouse gas emissions tied to its investments by up to 26% by restructuring its portfolios, moving investments away from greenhouse gas-intensive sectors. Many divestment campaigns have an additional “divest to reinvest” element that advocates using funds invested in fossil-fuel companies to reinvest in socially and environmentally beneficial projects, such as low-carbon and renewable schemes (99), creating the positive-feedback interactions with the STE1. An avalanche effect would be triggered if national banks and insurance companies warned against the global risk associated to stranded assets from fossil-fuel projects. These concerns are growing in Europe, and there are already signs of a tipping point, namely cuts in financial and insurance support for coal projects (100). Norwegian financial authorities might soon be divesting the country’s sovereign wealth fund. Around 6% (€30bn) of this fund’s wealth is invested in oil and gas companies (101).

#### STIs in the system of norms and values.

The extraction and use of fossil fuels out of line with the Paris Climate Agreement targets is arguably immoral, as it would cause widespread grave and unnecessary harm (97). The impact of greenhouse gas emissions disproportionately affects the most vulnerable social groups, such as women and children (102). It also affects the well-being of future human generations (103) and causes many direct negative health effects (104). Historical cases show that social and moral norms can affect human behavior on a large scale (38). The abolition of the transatlantic slave trade, for example, showed that changes in the ethical perception of slave labor at that time were consciously initiated by a small group of intellectuals (105). Revealing the moral implication of the continued burning of fossil fuels is an example of an intervention that is likely to induce a tipping process through changes in the human normative system, i.e., the system of moral and behavioral norms that influence what is rewarded and desired in the society. Norms can develop through social networks in neighborhoods or workplaces and support certain lifestyles or technology choices (106). A study on the installation of photovoltaic panels by home owners showed social networks and dwelling proximity explained the owners’ decision to install photovoltaic panels on their homes (107). The control parameter is represented by the ethical perception of fossil fuels, the environmental externalities they generate, and the broader harm they visit on societies. The critical condition in the control parameter will be achieved if the majority of social and public opinion leaders recognize the ethical implications of fossil fuels and generate pressure in their peer groups to ostracize the use of products involving fossil fuel burning. This could be more widespread in religious communities and be led by spiritual leaders, perhaps following the example of Pope Francis’s encyclical *Laudato si*’ (108). It could alternatively be manifested as a secular trend originating mainly from young, intellectually and social justice-oriented groups of people who might actively stand against supporters of fossil fuels—these would include extraction and utilization companies, governments supporting the latter, as well as the superrich family clans generating wealth from fossil fuel extraction and utilization in the last 150 y. The wealth of about 11% of the world’s billionaires is related to energy production (excluding solar and wind), mining, and other natural resource utilization (109). Recent experimental evidence shows that dominant social conventions or established behavior can be changed by committed minorities of roughly 25% of a group (36). Social norms are the sources of law (110); therefore, recognizing the immoral character of fossil fuels can further lead to regulations restricting the use and extraction of fossil fuels (111).

The time elapsing from the recognition of the activity as a problem and as a matter of a moral choice by international legal scholars, religious groups, and other moral entrepreneurs, to international delegitimization might range from a few decades to a few centuries. The slavery abolition movement started in 1772 in England and led to the abolition of the slave trade in 1807 and in the 1833 to the total abolition of slavery in the British Empire. The historical data show that although the number of slaves traded in the British Empire dropped to zero by 1826, the number of internationally traded slaves started to decrease around the mid-19th century. However, after reaching its peak, the number of slaves traded internationally decreased exponentially within just a few years. In the period 1851 to 1860, 71% fewer slaves disembarked than in the period 1841 to 1850 (https://slavevoyages.org/). A more recent example of outlawing the use of substances responsible for ozone depletion showed that such changes might occur in less than 30 y (112). However, the financial and political power of the fossil fuel industry suggests the need for much more substantial political effort to ensure such a change, than would have been the case for the issue of ozone depletion (99). There is recent anecdotal evidence that protests, such as the #FridaysForFuture climate strikes of school students around the world, the Extinction Rebellion protests in the United Kingdom, and initiatives such as the Green New Deal in the United States, might be indicators of this change in norms and values taking place right now (113).

#### STIs in the education system.

Many examples of research confirm the role of education in social transformations (114) and tackling climate change concerns (115, 116). The control parameter that relates to this intervention is the coverage of climate change issues in school and university teaching programs. While many teachers include some, often thin, coverage of climate change (117), comprehensive approaches at all levels of public education are still rare. Lack of knowledge about the causes, impacts, and solutions to climate change was the most easily identifiable individual barrier to engagement in climate action in the United Kingdom (118). At the same time, studies show that the divergent ways of understanding climate change draw on discourses broader than scientific knowledge; these differences may be blamed for misinterpretation of scientific notions such as uncertainty (119) as well as for the tendency to attribute responsibility for causing and mitigating climate change to others (118). Formal and lifelong education is traditionally considered a slow and evolving process, but there are examples of rapid change that can be generated. Quality education supports and amplifies norms and values and can quickly inspire behavior change among individuals and their cohorts. In addition, massive literacy campaigns, such as the one that took place in Cuba in the 1950s, where in a less than a year illiteracy was reduced from 24 to 3.9% (120), demonstrate the potential for rapid societal transformation. The effects of changes in educational programs can also lead to a social tipping process as soon as the new generation enters the job market and public decision-making bodies. The recent #FridaysForFuture protests demonstrate the upcoming new generation might radically change the political scene. It is estimated that within just half a year the school children movement grew to 1.5 million students in 125 countries. The effects of educational campaigns can be strengthened by a supportive family and community context as well as by media campaigns, advertising bans, higher taxes, use prohibitions, and lawsuits against producers (121). Warner (122) shows that combined educational and mass-media campaigns in the 1970s in the United States led to 4 to 5% annual decrease in cigarette consumption. In the climate change context, Dietz et al. (123) show that interventions that combine mass-media messages, household- and behavior-specific information, and communication through individuals’ social networks and communities could lead to reductions of 20% in household direct emissions in less than 10 y, with little or no reduction in household well-being. An educational campaign carried out in five Italian cities for 2 y, involving teachers, pupils, and citizens, resulted in an emission reduction in a range of 7 to 30% in the 247 families included in the research (124). That said, education to bolster understanding of the causes and effects of climate change, however important, will not be sufficient to transform society alone. Sustainability cannot be imposed, it has to be learned, so that is endogenously realized and enacted deliberately by the actors who constitute the SES (46). Engagement and the fostering of sustainable lifestyles and career pathways by transforming schools into living laboratories (125) is necessary to counter the often overlooked shadow side of education, since the secondary and higher levels of education are currently associated with higher resource use (126).

#### STIs through information feedbacks.

The last tipping intervention is related to the flow of information and creating positive information feedbacks. The control parameter is represented by the transparency of the impact of individual consumer and lifestyle choices and carbon emissions. Transparency and disclosure of information about carbon emissions are needed, for instance, not just to provide a solid basis for global, regional, and national policies (127) but also to increase public and consumer awareness and improve labeling programs (128), triggering action and lifestyle changes to support decarbonization (129). The recent disclosure of the close ties between RWE, the biggest energy company in Germany, and regional politicians protecting their interest in the lignite coal extraction areas in Hessen led to a nationwide social movement and massive public demonstrations against plans to clear the Hambach Forest (130). Corporate disclosure of carbon assets can also help to overcome the short-term horizons of fund managers (131) and create a positive feedback in the divestment movement.

Another positive feedback can be identified between the information system and public education. Enhanced public knowledge and understanding by the broader public of the main variables and processes in the Earth’s climate system and their linkages with human activities could increase public sensitivity to emissions-related information (132). Just as most product packages display nutritional facts, some authors propose they could display a second label on “Earth facts” and disclose the information on their carbon footprint and other emissions (133). In comparison, the global market for organic products, driven primarily by health concerns but clearly stimulated by providing clear labeling, increased at rates above 10% per year (134).

## Discussion and Conclusions

**Fig. 3.**
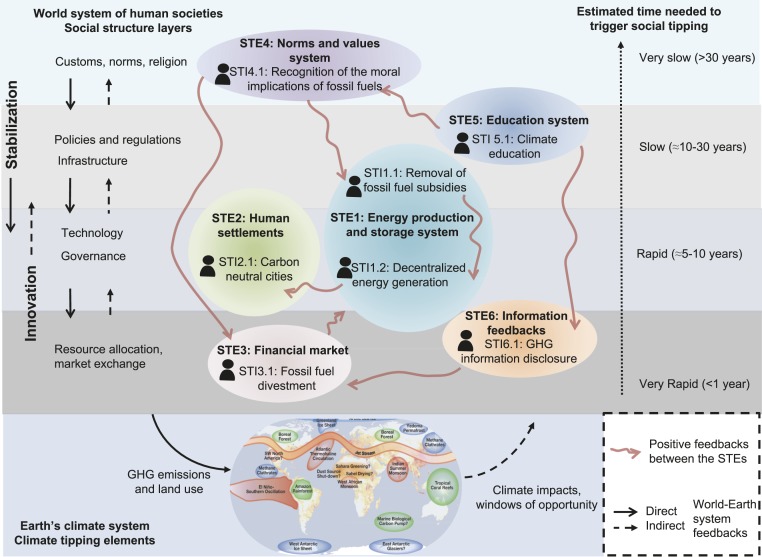
Social tipping elements (STEs) and associated social tipping interventions (STIs) with the potential to drive rapid decarbonization in the World–Earth system. The processes they represent unfold across levels of social structure on widely different timescales, ranging from the fast dynamics of market exchanges and resource allocation on subannual timescales to the slow decadal- to centennial-scale changes on the level of customs, values, and social norms (51).

Each of the STEs discussed above exists in the real world in varying degrees, locations, and scales and shows the potential to boost a decarbonization breakthrough. Since social-ecological dynamics are subject to complex processes that cannot be fully anticipated, it is not possible to predict when and where exactly tipping points will be crossed. However, the system can be imperfectly navigated intentionally to achieve certain desirable conditions and capacities (34). The social tipping dynamics are likely to spread through adaptive networks of interactions rather than via straightforward cause–effect systems. The identified interactions between the various STEs mean that they can potentially reinforce one another, making a transition to decarbonization more likely if several are triggered simultaneously (Fig. 3). In addition, crossing multiple tipping points in diverse systems of action increases the likelihood of breaking existing systemic inertia and lock-ins and thereby achieving the climate policy goals (34, 45). The interactions between the nominated candidates for STEs could be organized as different possible transformative pathways leading to crossing tipping points across scales and regions. These “tipping transformative pathways” can potentially show the bottom-up emergence of the global sustainability pathway (SSP1) (135).

One possible transformative pathway that has recently started to materialize has been initiated within the education system by school children who started the climate strikes #FridaysForFuture. The movement is causing “irritations” in personal worldviews (136) and thus might be changing peoples’ norms and values and the ways of thinking and acting, possibly leading to changes in policies and regulations, infrastructure development, as well as individual consumption and lifestyle decisions. For example, as a result of the massive school student protest in Germany, even the traditionally climate-conservative parties recently started to address climate change issues in their programs (137). The increasing awareness of the seriousness of climate change might drive an increasing demand for greenhouse-gas emission disclosure of various products and services. It might also drive an increased recognition of the intergenerationally unethical and immoral character of fossil fuels that will furthermore strengthen the legitimacy of carbon mitigation policies, including the removal of fossil-fuel subsidies. Although changes of norms, customs, and beliefs occur very slowly (138), one should keep mind that now is not year zero of the global sustainability transformation. It has now been 30 y since the Intergovernmental Panel on Climate Change (IPCC) was endorsed by the United Nations and issued its first report recognizing the anthropogenic character of climate change, and many important milestones have been reached since then, including publishing the subsequent IPCC reports, Pope Francis’s encyclical *Laudato si’*, and numerous events led by artists and activists increasing the concern about climate issues. The example of “flight shaming” that was initiated by a Swedish Olympic athlete and has been popularized in social media (139), shows that society may now be just at the edge of tipping in the realm of social norms and beliefs. The high number of seats that environmentally oriented parities recently won in the European Union (EU) elections (140) shows that EU policy might potentially undergo a substantial shift within the next few years, the EU becoming a global leader in carbon mitigation efforts.

A global breakthrough could also be initiated at the level of resource allocation by redirecting financial flows in line with the divestment movement and improving information feedbacks by disclosing the greenhouse gas emissions of products and services. At this level, firms take consumption and production decisions constrained by budget as well as by information and technology availability (20, 51). Changes at this level occur continuously. Very rapid changes, at a rate of 50% or more, can occur within a few months. This is shown by public opinion polls on, for example, political preferences following information flows, particularly in online social media (141). Rapid changes in stock markets can occur within hours (142). Nevertheless, such trends rarely lead to bigger changes in human societies without simultaneous institutional changes. The institutional changes, requiring more time, such as transforming the public subsidies and taxation systems, are needed to stabilize the new emerging system. Otherwise the system might become increasingly unstable, bouncing back and forth between the old and new social order, delaying the transformation. A well-documented example of such a phenomenon is the rebound effect (143, 144). Even the frequently quoted “successful” example of feed-in tariffs and German energy transition “Energiewende” to renewables, which used the rapid change in public opinion in the aftermath of the nuclear catastrophe in Fukushima in Japan in 2011, have recently faded away due to the lack of sufficiently sustained societal and policy support (145).

Many of the nominated candidates for STEs extend beyond achieving greenhouse gas reduction and can be potentially interlinked with achieving other global policy goals, such as the Sustainable Development Goals. Many of the interventions discussed above include a range of well-being and public health cobenefits (68). Solving the climate crisis could be a chance to redesign the global socioeconomic institutions toward achieving a more just and equitable future (146). Several authors point out that environmental catastrophes, including increased severity and frequency of climatic extremes, might act as “windows of opportunity” that give rise to uncertainty and confusion, which might in turn motivate actors to engage in reflective processes and take sharp breaks from the existing procedures and policies (147) (Fig. 3). However, although the opportunity for a revolutionary change might emerge due to external or environmental factors (148), it is important to actively work with the social complexities (Fig. 2) and the relevant key social actors (Tables 2 and 3), to increase public acceptance and support for the transformative changes to come. To ensure that climate-related social learning will take place, it is necessary to understand how changes of perceptions and awareness, motives, and interests of various actors take place and how institutional innovations occur (149).

We call on both social and natural sciences to engage more intensively in collaborative interdisciplinary research to understand rapid social transformations, STEs, and their interactions with tipping elements in the Earth system. Planetary social-ecological models and machine-learning techniques can help to explore the control parameters and critical thresholds in the trajectory of this World–Earth coevolutionary dynamics (43). We also encourage studies on the archetypes of social transformations (150) in different world regions as well as using insights and methods from the natural sciences to study the complexity of social systems. Both empirical studies and modeling exercises could also help to assess the distributional impacts of STIs and factors influencing their effectiveness. Our study presents a comprehensive empirical analysis of social tipping dynamics for global decarbonization. However, since our results were derived from an elicitation process involving small and nonrepresentative samples of experts, more research is needed to verify our findings and to provide more robust empirical evidence and data. Experts from the research sector and the Global North were overrepresented in our sample. Therefore special attention should be given to the expertise of low-carbon and sustainability practitioners as well as to providing more empirical material from the Global South. Finally, the urgency and complex character of climate change require transdisciplinarity and engagement with social movements, knowledge brokers, and change leaders (151). More research is needed on understanding the required social processes and the drivers and incentives for short-term engagement of diverse coalitions of action around concrete solutions and strategies at various governance levels (152).

## Materials and Methods

The primary data collection tool was an online expert survey that was sent to over 1,000 international experts through a private message or addressed through mailing lists of organizations in the field of climate change and sustainability. A full list of all survey questions as well details on the research organization are provided in *SI Appendix*. The survey ran for 2.5 mo, and it was completed by 133 experts. In total, they suggested 207 candidates for STEs and interventions instrumental for decarbonization by 2050. A selected group of 17 experts were invited for a workshop that focused on choosing the top candidates for STEs. Finally, the coauthors carried out a literature review on the top candidates selected at the workshop, following the literature review guidelines.

### Data Availability Statement.

All data discussed in the paper will be made available to readers upon request.

## Supplementary Material

Supplementary File
