# Supplementary material for: Social tipping dynamics for stabilizing Earth’s climate by 2050
Source: Proc Natl Acad Sci U S A. 2020 Jan 21;117(5):2354–65. doi: 10.1073/pnas.1900577117 (PMC7007533; doi:10.1073/pnas.1900577117)
Supplement: Supplementary File [file pnas.1900577117.sapp.pdf]

Social tipping dynamics for stabilizing Earth's climate by 2050

Supporting information

Table of content

Organization of the expert elicitation process……………………………………………………………………2

Formal definition and filtering of social tipping elements for decarbonization that was used in the literature review……6

Online expert survey ………………………………………………………………………………………………………..8

www.pnas.org/cgi/doi/10.1073/pnas.1900577117

Organization of the expert elicitation process

Expert Survey

The primary data collection tool was an online expert survey that was sent to over 1000 international experts through a private message or addressed through mailing lists of organizations in the field of climate change and sustainability. The online survey contained a welcome page explaining the research objectives and presetting the research team, a page with definitions and terminology used in the research, the main survey page where we asked for the description of the candidate for social tipping element, that was followed by a page gathering personal information such as self-assed expertise level, field of work, residence country, gender and age. The final page contained a lottery where all experts who completed the survey could win a book. The survey run for 2.5 months starting mid-September 2016 and was closed at the end of November 2016.

In the main part of the survey, the survey participants were asked to name and describe the candidate for the social tipping elements, explain the tipping mechanism, as well as the geographical and governance level where it might be occurring, relevant actors, and estimate their confidence level that this social tipping element will be actually occurring and leading to a decarbonization by 2050. The experts could nominate up to 6 candidates for social tipping elements and the answers could be supported by scientific references.

The main continent questions asked in the survey included:

1. What is the most important social tipping element that could initiate a socially and economically disruptive transformation leading to a complete decarbonization by 2050? The "social" component of the term is used very broadly, and could for example refer to policies, economic mechanisms, technologies and social value changes.
2. (Optional) What is the relevant geographical region, governance scale and/or socio-economic sector where the social tipping element would occur?
3. (Optional) Who are the relevant actors (e.g. the public, government, industry) that could play a role in leveraging the tipping element to a larger scale?
4. (Optional) How confident are you that the associated social tipping point is actually going to take place and contribute substantially to a rapid and complete global decarbonization by 2050?

In addition, the survey included questions about the self-expertise assessment of the experts and the background (education level, field of expertise, type of sector) and personal information (gender, age, the country of residence).

The online survey was completed by 133 experts. In total they suggested 207 candidates for social tipping elements. Five answers that were either in contrary with achieving SDG (i.e. killing majority of human population) or located outside the human agency (alien intervention, terminal disease spread) were excluded from further analysis. Nine percent of the experts ranked themselves as leading experts in the field, nearly 39% as one having a specialized expertise on the topic of the survey, 26% as having a general expertise, 19.5% stated that the topic of the survey is related to their expertise and finally 7% admitted that the topic of the survey is not one of areas of their expertise. Nearly 80% of the experts were representing various research sectors, 9% business and industry, 8%

NGOs and civil organizations, 3% governmental organizations. Fifty six percent of respondents declared Europe as their primary place of residence and work, for 19% it was North America, for 13% Asia, 4% lived in Africa, and 2% in Australia and New Zealand. Most of the survey experts were male (71%), and most of the experts who took part in the research were in the age group of 30-39 years old (31%) or 40-49 years old (28%).

The length of experts' responses and social tipping element descriptions varied from using just a few words to answers reaching over 4,400 words. Although the definition of social tipping elements was provided to the survey participants, some experts who took part in the research did not follow the definition, and provided examples of particular interventions leading to social tipping rather than naming and describing the social tipping elements. Using qualitative data analysis we grouped the answers in twelve basic categories of social tipping elements presented in Table 2 in the manuscript.

Expert Workshop

A selected group of 17 experts were invited to take part in an expert workshop that focused on choosing the top candidates for social tipping elements instrumental for decarbonization by 2050, differentiating the tipping interventions, key actors, and assessing the interactions between the social tipping elements. The workshop took place in December 2016 at the Stockholm Resilience Center in Sweden. The Stockholm Resilience Center covered the costs of workshop attendance of those workshop participants who reported to have problems with travel funding at their home organizations. The workshop participants received a shortened version of the expert survey answers one week before the workshop. The workshop resulted in choosing eight top candidates for social tipping elements and 10 candidates for social tipping interventions.

Table SI1: Invited expert workshop participants

Name	Affiliation	Sector / type of work	Country
Ilona M. Otto	Potsdam Institute for Climate Impact Research	Research	Germany and Poland
Johan Rockström	Stockholm Resilience Center	Research	Sweden
Jonathan F. Donges	Potsdam Institute for Climate Impact Research	Research	Germany
Avit K. Bhowmik	Stockholm Resilience Center	Research	Sweden and Bangladesh
Roger Cremades	Climate Service Center	Research	Germany and Spain
Franziska Allerberger	Graz University	Master student	Austria
Wolfgang Lucht	Potsdam Institute for Climate Impact Research	Research	Germany
Richard Hewitt	James Hutton Institute	Research	UK
Jennifer Hinton	Stockholm Resilience Center	Research	Sweden and USA
Mark McCaffrey	Centre for Sustainable Development Studies, National University for Public Service	Research	Hungary
Detlef van Vuuren	BL Netherlands Assessment Agency	Research / Policy	The Netherlands
Nerea Moran	University of Madrid	Research	Spain
Alex Lenferna	University of Washington	PhD student	USA
James Greyson	BlindSpot Think Tank	NGO	UK
Yuyun Ismawati*	Balifocus	NGO	Indonesia
Sylvanus Doe	Geospecies Consult	NGO	Ghana
Benoit Faraco	European Climate Foundation	Policy	France

* Did not show up at the workshop due to unexpected travel difficulties

Table SI2: Candidates for social tipping elements and social tipping interventions selected by the workshop participants.

Social tipping element	Social tipping intervention
Norms and value system	Recognizing the immoral character of fossil fuels
Knowledge system	Transformative narrative emphasizing the benefits to be gained by decarbonization;<br>Developing alternative welfare measurements
Education system	Climate education and engagement
Energy production and storage	Subsidy programs;<br>Decentralized energy generation
Human settlements	Carbon-neutral cities
Citizenship involvement	A global network of social movements
Financial markets	Divestment movement
Information feedbacks	Emission information disclosure

Literature review guidelines

The selected by the workshop participants top candidates for social tipping elements and tipping interventions were furthermore analyzed in a literature review process that aimed at finding out empirical examples demonstrating the potential to reduce the greenhouse gas emissions by the nominated candidates for social tipping interventions as well as estimating the time needed to trigger the social tipping within the distinguished candidates for social tipping elements. The workshop participants were given a chance to contribute to the literature review. Those who positively responded and submitted the literature review on the selected candidates for social tipping interventions joined the paper author team. The literature review resulted in differentiating the final set of social tipping elements and social tipping interventions presented in the manuscript (Tab. 3 in the manuscript).

The literature review guidelines included the following instruction:

Please write about 1 page input on the social tipping element that you lead. Before you start, please read the formal definition of social tipping elements. Please structure the literature review according to the following topics:

1. A brief description of the social tipping element candidate.
    a. What is the key intervention of interest?
    b. Why is this social tipping element relevant for a rapid decarbonization? How can this relevance be measured or judged?
    c. What are the parameter(s) projecting onto the control ρ, and their value(s) near ρ_{crit}? I.e. is there an observable and quantifiable/measurable threshold or evidence from it on the literature?
    d. Is the social tipping element related behavior observable and can it be imitated?
    e. Where (region) and on which scale does it occur (local, national, regional, global)?

2. What is the mechanism of tipping?
    a. Who are the relevant actors and how do they interact? Does the social tipping element provide (co-)benefits for those actors involved?
    b. Is there a self re-inforcing accelerating feedback implying that a small change in the subsystem can lead to big impacts on the whole system?
    c. What are the involved variables and processes?
3. What is the time needed to trigger the tipping process (human intervention/decision-making time T_H) and what is the time needed to complete the tipping process and to observe the qualitative change in the system (transition time T)?
4. Are there other related factors that amplify the effect of this STE on decarbonization (interaction with other tipping elements)?
5. What are the associated uncertainties specifically regarding the critical threshold value and time scales?

Formal definition and filtering of social tipping elements for decarbonization that was used in the literature review

Motivation and filtering

The elicitation as evaluated by our STE workshop provided a **list of candidate social tipping elements** for decarbonization. This list now has to be **filtered, using 2 criteria**

1. Is there a scientific literature that allows to put the argument on the social tipping element on a solid footing (not scientific consensus needed, just a credible case)?
2. Does the social tipping element candidate fulfill the criteria of a formal definition (see below) of what an STE is (as opposed to some other important social-economic process)?

Formal definition

To be considered a social tipping element relevant for decarbonization, a candidate Σ must fulfill the following criteria based on the definition by John Schellnhuber of tipping elements used in Lenton et al. (2008):

1. (**Existence of a tipping point**) The parameters controlling the social-economic subsystem Σ can be transparently combined into a single control ρ, and there exists a critical control value ρ_{crit} from which any significant variation by $\delta\rho > 0$ leads to a qualitative change ($\hat{F}$) in a crucial system feature F, after some observation time $T > 0$, measured with respect to a reference feature at the critical value, i.e.,

$$|F(\rho \geq \rho_{\text{crit}} + \delta\rho|T) - F(\rho_{\text{crit}}|T)| \geq \hat{F} > 0. \qquad [1]$$

   This inequality applies to forcing trajectories for which a slight deviation above a critical value that continues for some time inevitably induces a qualitative change. This change may occur immediately after the cause or much later. The definition encompasses equilibrium properties with threshold behavior as well as critical rates of forcing. In its equilibrium application, it includes all orders of phase transition and the most common bifurcations: saddle-node and Hopf bifurcations.

   Here we restrict ourselves to social tipping elements that may be accessed by human agency though a specific human intervention. We define the subset of relevant social tipping elements for decarbonization by adding to condition 1 the following *normative* conditions:

2. (Human **intervention time horizon**) Human activities are interfering with the system Σ such that decisions taken within a “human intervention time horizon” ($T_H > 0$) can determine whether the critical value for the control ρ_{crit} is reached. This occurs at a critical time (t_{crit})

that is usually within T_H but may be later because of a commitment to further change made during T_H.

3. (**Decarbonization time horizon**) The time to observe a qualitative change plus the time to trigger it lie within a "decarbonization time horizon" (T_D); $t_{crit} + T \leq T_D$. T_D recognizes that events manifesting too far away in the future are not relevant for achieving the goal of rapid decarbonization of the global social-economic system.
4. (**Relevance for decarbonization**) The qualitative change in system Σ contributes significantly to decarbonization of the global social-economic system. A qualitative change should correspondingly be defined in terms of impacts such as expected emissions reductions.

Relevant time horizons for filtering STEs

- **Human intervention time horizon**: T_H = 1 – 10 years, since time until 2050 is short.
- **Decarbonization time horizon**: T_D is determined by the goal of full decarbonization of the global social-economic system by 2050. Since we are in 2017 now, T_D = 33 years.

Questions for literature review arising from this definition

- What are the potentially relevant social tipping elements for decarbonization?
- What is the specific human intervention activating the tipping mechanism?
- And for each social tipping element:
    - What is the mechanism of tipping?
    - What is the key feature *F* of interest?
    - What are the parameter(s) projecting onto the control ρ, and their value(s) near ρ_{crit}?
    - How long is the transition time *T*?
    - What are the associated uncertainties?

References

Lenton, T. M., Held, H., Kriegler, E., Hall, J. W., Lucht, W., Rahmstorf, S., & Schellnhuber, H. J. (2008). Tipping elements in the Earth's climate system. *Proceedings of the National Academy of Sciences USA*, *105*(6), 1786-1793.

Fragebogen

1 Welcome

Questionnaire for experts

Disruptive social tipping elements for a rapid decarbonization

If the Sustainable Development Goals and the Paris Climate Agreement are taken seriously, then a global sustainability transition could be achieved in the next 20 years and take us to a more equitable and just global societey operating within the planetary boundaries. The central prerequisite for such a global sustainability transition is rapid decarbonization. By decarbonization we mean a complete transition in the energy system away from carbon-intensive fossil fuels[1]. While many researchers are focusing on incremental changes and the gradual phasing out of coal, we would like to identify social processes and leverages that could cause *abrupt changes triggering rapid decarbonization.*

We are asking you to nominate candidates for social tipping elements that could lead to a disruptive change and a rapid emergence of a new type of a fossil-free economy.

Before you start the survey it is useful to be clear about the concept and terminology. Similarly to Lenton and his co-authors[2], we use the term tipping element to describe a subsystem where a small change or innovation can tip the system into a qualitatively different state. Our understaning of the "social" component is very broad and could refer to social, economic and political mechanisms as well as to technologies that could substantially change the socio-ecological relationships. A social tipping element could be for example a policy that shuts down coal power plants or stops production of new cars that run on fossil fuels, or a social value shift that would lead to a rapid decarbonization. Our research has an explorative character and it is one of our goals to clarify the meaning of social tipping elements. Here is a graphical illustration of the concept:

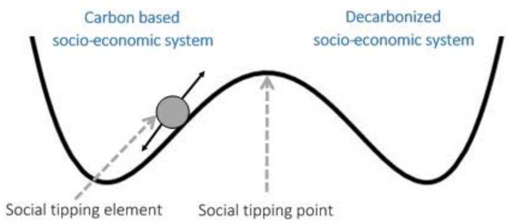

The questionnaire has only five pages. At the end of the questionnaire you will be asked whether you are interested in taking part in a workshop that will synthesize the findings from this questionnaire and that will take place in fall 2016 at the Stockholm Resilience Center, Sweden. On the last page there will also be a chance to win a book.

We follow common data protection policies and, hence, your personal information will remain anonymous. Providing your name and your full institutional affiliation is optional. If you want to go back to the previous pages during filling in the questionnaire and keep the text that you entered, please use the "Back" button located in the left bottom corner of each page.

With best regards,

Johan Rockström and the Earth League.

Research team:
Ilona. M. Otto
Jonathan F. Donges
Franziska Allerberger

In case of problems and questions please contact ilona.otto@pik-potsdam.de

References

1. Fisher, B.S., N. Nakicenovic, K. Alfsen, et al. (2007) Issues related to mitigation in the long term context, In: Climate Change 2007: Mitigation. Contribution of Working Group III to the Fourth

Assessment Report of the Intergovernmental Panel on Climate Change, Cambridge University Press, Cambridge.

2. Lenton, T.M., et al. (2008) Tipping elements in the Earth's climate system, PNAS 105(6): 1786-1793.

2 Question 1

Page 1.A

1. What is, in your opinion, the most important social tipping element that could initiate a socially and economically distruptive transformation leading to a complete decarbonization by 2030?

The "social" component of the term is used very broadly, and could for example refer to policies, economic mechanisms, technologies and social value changes.

Briefly explain why you have chosen this tipping element, and state your arguments why it could induce a rapid decarbonization. If possible, please include the references and links to the relevant sources. The subsequent questions will ask for more specific characteristics. **Please describe only one social tipping element on this page. When you click the "Continue" button, you will have the option to suggest other tipping elements.**

2. What is the relevant geographical region, governance scale or socio-economic sector where the social tipping element would occur?

3. Who are the relevant actors (e.g. the public, government, industry) that could play a role in leveraging the tipping element to a larger scale?

4. Can a threshold value of the associated tipping point be estimated, e.g. a certain percentage of market share that has to be achieved for a new technology to take off?

If relevant, please provide the minimum and maximum estimate and/or the uncertainty range.

5. How confident are you that the described phenomenon is indeed a social tipping element that could lead to a rapid and complete global decarbonization by 2030?

Please select the option.

- Very uncertain
- Uncertain
- Rather uncertain
- Rather confident
- Confident
- Very confident

6. How confident are you that the associated social tipping point is actually going to take place and lead to a rapid and complete global decarbonization by 2030?

Please select the option.

- Very uncertain
- Uncertain
- Rather uncertain
- Rather confident

- Confident
- Very confident

2.1 Tipping element 2

Page 2.A
Would you like to suggest one more social tipping element?

Please select the option.

- Yes
- No

3.1 Filter

Page 1.B
1. In your opinion, what is the second most important social tipping element for a rapid and complete decarbonization by 2030?

Briefly explain why you have chosen this tipping element, and state your arguments why it could induce a rapid decarbonization. If possible, please include the references and links to the relevant sources. Please describe only one social tipping element on this page. When you click the "Continue" button, you will have the option to suggest other tipping elements.

2. What is the relevant geographical region, governance scale or socio-economic sector where the social tipping element would occur?

3. Who are the relevant actors (e.g. the public, government, industry) that could play a role in leveraging the tipping element to a larger scale?

4. Can a threshold value of the associated tipping point be estimated, e.g. a certain percentage of market share that has to be achieved for a new technology to take off?

If relevant, please provide the minimum and maximum estimate and/or the uncertainty range.

5. How confident are you that the described phenomenon is indeed a social tipping element that could lead to a rapid and complete global decarbonization by 2030?

Please select the option.

- Very uncertain
- Uncertain
- Rather uncertain
- Rather confident
- Confident
- Very confident

6. How confident are you that the associated social tipping point is actually going to take place and lead to a rapid and complete global decarbonization by 2030?

Please select the option.

- Very uncertain
- Uncertain
- Rather uncertain

- Rather confident
- Confident
- Very confident

4.1 Filter Tipping point 3

Page 2.C
Would you like to suggest one more social tipping element?

Please select the option.

- Yes
- No

5.1 Filter

Page 1.C
1. In your opinion, what is the third most important social tipping element for a rapid and complete decarbonization by 2030?

Briefly explain why you have chosen this tipping element, and state your arguments why it could induce a rapid decarbonization. If possible, please include the references and links to the relevant sources. Please describe only one social tipping element on this page. When you click the "Continue" button, you will have the option to suggest other tipping elements.

2. What is the relevant geographical region, governance scale or socio-economic sector where the social tipping element would occur?

3. Who are the relevant stakeholders (e.g. the public, government, industry) that could play a role in leveraging the tipping element to a larger scale?

4. Can a threshold value of the associated tipping point be estimated, e.g. a certain percentage of market share that has to be achieved for a new technology to take off?

If relevant, please provide the minimum and maximum estimate and/or the uncertainty range.

5. How confident are you that the described phenomenon is indeed a social tipping element that could lead to a rapid and complete global decarbonization by 2030?

Please select the option.

- Very uncertain
- Uncertain
- Rather uncertain
- Rather confident
- Confident
- Very confident

6. How confident are you that the associated social tipping point is actually going to take place and lead to a rapid and complete global decarbonization by 2030?

Please select the option.

- Very uncertain
- Uncertain

- Rather uncertain
- Rather confident
- Confident
- Very confident

6.1 Filter Tipping point 3

Page 2.D
Would you like to suggest one more social tipping element?

Please select the option.

- Yes
- No

7.1 Filter

Page 1.D
1. In your opinion, what is the fourth most important social tipping element for a rapid and complete decarbonization by 2030?

Briefly explain why you have chosen this tipping element, and state your arguments why it could induce a rapid decarbonization. If possible, please include the references and links to the relevant sources. Please describe only one social tipping element on this page. When you click the "Continue" button, you will have the option to suggest other tipping elements.

2. What is the relevant geographical region, governance scale or socio-economic sector where the social tipping element would occur?

3. Who are the relevant actors (e.g. the public, government, industry) that could play a role in leveraging the tipping element to a larger scale?

4. Can a threshold value of the associated tipping point be estimated, e.g. a certain percentage of market share that has to be achieved for a new technology to take off?

If relevant, please provide the minimum and maximum estimate and/or the uncertainty range.

5. How confident are you that the described phenomenon is indeed a social tipping element that could lead to a rapid and complete global decarbonization by 2030?

Please select the option.

- Very uncertain
- Uncertain
- Rather uncertain
- Rather confident
- Confident
- Very confident

6. How confident are you that the associated social tipping point is actually going to take place and lead to a rapid and complete global decarbonization by 2030?

Please select the option.

- Very uncertain

- Uncertain
- Rather uncertain
- Rather confident
- Confident
- Very confident

8.1 Filter Tipping point 3

Page 3.E
Would you like to suggest one more social tipping element?

Please select the option.

- Yes
- No

9.1 Filter

Page 1.E
1. In your opinion, what is the fifth most important social tipping element for a rapid and complete decarbonization by 2030?

Briefly explain why you have chosen this tipping element, and state your arguments why it could induce a rapid decarbonization. If possible, please include the references and links to the relevant sources. Please describe only one social tipping element on this page. When you click the "Continue" button, you will have the option to suggest other tipping elements.

2. What is the relevant geographical region, governance scale or socio-economic sector where the social tipping element would occur?

3. Who are the relevant actors (e.g. the public, government, industry) that could play a role in leveraging the tipping element to a larger scale?

4. Can a threshold value of the associated tipping point be estimated, e.g. a certain percentage of market share that has to be achieved for a new technology to take off?

If relevant, please provide the minimum and maximum estimate and/or the uncertainty range.

5. How confident are you that the described phenomenon is indeed a social tipping element that could lead to a rapid and complete global decarbonization by 2030?

Please select the option.

- Very uncertain
- Uncertain
- Rather uncertain
- Rather confident
- Confident
- Very confident

6. How confident are you that the associated social tipping point is actually going to take place and lead to a rapid and complete global decarbonization by 2030?

Please select the option.

- Very uncertain
- Uncertain
- Rather uncertain
- Rather confident
- Confident
- Very confident

10.1 Filter Tipping point 3

Page 2.F
Would you like to suggest one more social tipping element?

Please select the option.

- Yes
- No

11.1 Filter

Page 1.F
If you would like to suggest more social tipping elements, please type and describe them shortly here.

12.1 Experitise evaluation

Page 3
How would you evaluate your expertise in the area of decarbonization and social transformation?

Please select one of the options.

- One of leading experts
- An expert
- Rather an expert
- Rather not an expert
- Not an expert

13 Test_Personal_Questions_mixextern_2

Page 4
1. Your name (optional)

If you don't want to share your name, please leave this field empty.

2. Your affilation or employing organization (optional)

If you don't want to share your affiliation, please leave this field empty.

3. What sector are you working for?
Please choose from the options below and specify if necessary.

- Government, politics and policy
- Other public sector
- Research (please specify)
  - Please select one of the options
  - Humanities
  - Performing or visual arts
  - Social sciences
  - Economics
  - Biological and medical sciences
  - Earth and environmental science
  - Physical sciences
  - Engineering and technology
  - Engineering and technology
  - Mathematics, statistics and infor
  - Interdisciplinary research
- NGO (please specify)
  - Please select one of the options
  - Civil society
  - Development
  - Environment

- Business (please specify)
  - Please select one of the options
  - Food, water and agriculture
  - Finance, banking, economics
  - Energy and resources
  - Construction and infrastructure
  - Health and wellbeing
  - Manufacture
  - Transport
  - Chemicals, technology
  - Telecommunication, media, ente
- Other

3. What sector are you working for? (obligatory)
Please select from the options below.

- Research
- Humanities
- Performing or visual arts
- Social Sciences
- Economics
- Biological and medical sciences
- Earth and environmental sciences
- Physical sciences
- Engineering and technology
- Mathematics, statistics, information sciences
- Interdisciplinary research

- Business (please specify from list)
  - Please select one of the options
- Government, politics and policy
- Other public sector
- NGO (please specify from list)
  - Please select one of the options
- Other

4. What is your highest education degree? (obligatory)
Please select one of the options.

- PhD, equivalent or higher
- Master or equivalent
- Bachelor or equivalent
- Secondary education or lower

5. What was the field of your higher level studies? (obligatory)
Please select one of the options.

- Humanities
- Performing or visual arts
- Social sciences
- Economics
- Biological and medical sciences
- Earth and environmental sciences
- Physical sciences
- Engineering and technology
- Mathematics, statistics, information sciences
- Interdisciplinary research

Other

6. For how many years are you employed since the award of your highest qualification?
Please select one of the options.

Please select one of the options

7. What is the country of your residence?
Please type your answer below.

7. What is the country of your residence? (obligatory)
Please select one of the options.

Please select country
Afghanistan
Åland Islands
Albania
Algeria
American Samoa
Andorra
Angola
Anguilla
Antarctica
Antigua and Barbuda
Argentina
Armenia
Aruba
Australia
Austria
Azerbaijan
Bahamas
Bahrain
Bangladesh
Barbados
Belarus
Belgium
Belize
Benin
Bermuda
Bhutan
Bolivia
Bosnia and Herzegovina
Botswana
Bouvet Island
Brazil
British Indian Ocean territory
Brunei Darussalam
Bulgaria
Burkina Faso
Burundi
Cambodia
Cameroon
Canada
Cape Verde
Cayman Islands
Central African Republic
Chad
Chile
China
Christmas Island
Cocos (Keeling) Islands
Colombia
Comoros
Congo
Congo, Democratic Republic
Cook Islands
Costa Rica
Côte d'Ivoire (Ivory Coast)
Croatia (Hrvatska)
Cuba
Cyprus
Czech Republic
Denmark
Djibouti
Dominica
Dominican Republic
East Timor
Ecuador
Egypt
El Salvador
Equatorial Guinea
Eritrea
Estonia
Ethiopia
Falkland Islands
Faroe Islands
Fiji
Finland
France
French Guiana
French Polynesia
French Southern Territories
Gabon
Gambia
Georgia
Germany
Ghana
Gibraltar
Greece
Greenland
Grenada
Guadeloupe
Guam
Guatemala
Guinea
Guinea-Bissau
Guyana
Haiti
Heard and McDonald Islands
Honduras
Hong Kong
Hungary
Iceland
India
Indonesia
[illegible]

8. What is your age?
Please select one of the options below.

Please select one of the options
18-29
30-39
40-49
50-59
60 or older
I prefer not to disclose

9. What is your gender?
Please select one of the options below.

Please select one of the options
Female
Male
I prefer not to disclose

10. Are you interested in taking part in the synthesis workshop that will take place in fall 2016 at the Stockholm Resilience Center?
Please select one of the options below.

- No
- Yes - please provide the e-mail address to which we could send a workshop invitation

11. Do you have any comments or feedback on our research?
Please type your comments below.

14 Lottery

Page 5 (the last one!)

1. Would you like to take part in a lottery and have a chance to win a copy of one of the books signed by the authors:

"Selbstverbrennung" by John Schellnhuber:

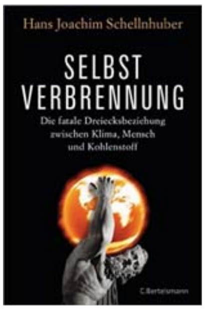

"Big World Small Planet" by Johan Rockström:

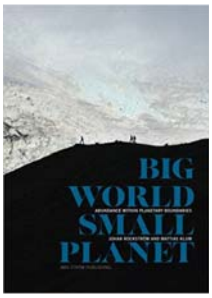

Please select one of the options

- No, I don't want to take part in the lottery.
- Yes, I would like to have a chance to win "Selbstverbrennung", here is my e-mail address to which you could send a notification if I win the book.
- Yes, I would like to have a chance to win "Big World Small Planet", here is my e-mail address to which you could send a notification if I win the book.

2. Are you interested in getting a copy of the report presenting these research results?

Please select one of the options

- No
- Yes, I would like to get a copy of the research report, here is my e-mail address to which you can send it.

15 Endseite

Thank you for taking our survey. We appreciate your time and expertise.

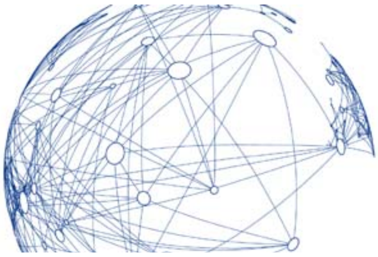

THE EARTH LEAGUE

Towards a Global Research & Assessment Alliance
